# Research Progress Regarding Interfacial Characteristics and the Strengthening Mechanisms of Titanium Alloy/Hydroxyapatite Composites

**DOI:** 10.3390/ma11081391

**Published:** 2018-08-09

**Authors:** Feng Li, Xiaosong Jiang, Zhenyi Shao, Degui Zhu, Zhiping Luo

**Affiliations:** 1 School of Materials Science and Engineering, Southwest Jiaotong University, Chengdu 610031, China; lifengswjtu@yeah.net (F.L.); dgzhu@home.swjtu.edu.cn (D.Z.); 2 Department of Chemistry and Physics, Fayetteville State University, Fayetteville, NC 28301, USA; zluo@uncfsu.edu

**Keywords:** Ti/HA, interface characteristic, biocompatibility, mechanical properties, strengthening mechanism

## Abstract

Titanium alloy/Hydroxyapatite (HA) composites have become a hot research topic in biomedical materials, while there are some challenges concerning bioactivity and mechanical properties such as low interface adhesion at the interface between metal and ceramic, complex interfacial reactions, and so on. Nevertheless, composites with reinforced phases can reach special properties that meet the requirements of biomedical materials due to the strong interfacial interactions between reinforcing phases (nano-carbon, partial oxides, and so on) and Titanium alloys or HA. This review summarizes the interface properties and mechanisms of Titanium alloy/HA composites, including interfacial bonding methods, strengthening and toughening mechanisms, and performance evaluation. On this basis, the interface characteristics and mechanisms of the Titaniumalloy/HA composites with enhanced phase are prospected. The results show that the interfacial bonding methods in the Titanium alloy/HA composites include chemical reactions and mechanical effects. The strengthening and toughening mechanisms contain grain refinement strengthening, second phase strengthening, solution strengthening, cracks and pulling out mechanisms, etc. This review provides a guidline for the fabrication of biocomposites with both mechanical properties and bioactivity.

## 1. Introduction

Recently, as bone grafting for humans has increasingly challenged the emergence of various diseases and disaster accidents, the development of biological transplantation materials has helped to repair or replace human tissues to improve their performance activities [1]. Since the 1960s, Titanium alloys have been widely used in medical implants and biomaterials due to their excellent corrosion resistance, abrasion resistance, strength, and machinability [2,3]; their non-toxicity and mechanical properties can no longer meet the current standards for biomaterials, and their biological performance is critical to promoting human tissue growth for biomaterials [3]. Pure metal graft materials, such as Titanium alloys, do not meet human needs due to their own lower bioactivity. Hydroxyapatite (HA), whose composition and structure are similar to those of human bone, can promote human tissue growth with higher biological compatibility, while its weak mechanical properties limit its use [4,5,6,7,8]. The comparison of the mechanical properties between HA, Titanium alloy, and composites can been seen in Table 1. Therefore, the composites prepared by Titanium alloy and HA can achieve a balance between mechanical properties and biological activities to a certain extent using plasma electrolysis oxidation, micro-arc oxidation, and other technologies [9,10,11,12,13,14]. Rafieerad et al. prepared HA coating on Titanium alloy by plasma electrolysis oxidation [15]. Their results showed that HA enhances high temperature adaptability and hardness of the composite. The porous layer generated on the surface of HA was helpful to the bioactivity of the composites [15]. Similar conclusions are reported elsewhere [16]. Yang et al. found a thick apatite layer on the surface of coated HA/Ti composite compared with substrate alloy, suggesting the improvement of bioactivity [17].

However, there are some main defects concerning the interface between the substrate and the coating in the coated materials, such as lower fracture toughness of HA, interface cracks in the deposition process, low interface adhesion at the interface between metal substrate and the ceramic, and uneven thickness distribution [9,13,14,17,18]. As an example, triclinic apatite coating on the Titanium substrate prepared by chemical deposition gradually occurred during plastic deformation, which separated from the matrix when a load was continuously improved. This phenomenon of interface debonding was a common failure mode [19]. However, uncoated composites prepared by powder metallurgy can effectively avoid existing problems at the metal substrate-coating interfaces. It was found that HA can be uniformly distributed in Titanium alloy matrix and was linked tightly with the matrix in a Ti-6Al-4V/HA composite prepared by vacuum hot-pressing, without the problem of the weak interface combination between the metal matrix and ceramic [20]. Other researchers showed that the hardness and density of Ti-Nb-Mo-CPP biocomposite prepared by spark plasma sintering (SPS) improved when the milling time was increased [21]. The density of Ti-6Al-7Nb/HA composite prepared by selective laser melting was found to improve with the increase of laser energy, which resulted in the increase of tensile strength [22]. The microstructure of composites was affected by local heating in the preparing process, such as the appearance of black zonal substances and columnar grains due to chemical isolation [22]. Some diverse bone apatite layers were found to spontaneously nucleate and precipitate on the surface of (Ti-13Nb-13Zr)/10HA composites prepared by SPS, which revealed their excellent biological activity [23]. However, there are some problems such as stress shielding phenomenon, complex reactions, and so on [1,4,5,6]. Research showed that there were some new phases such as Ca_5_(PO_4_)_3_(OH), CaTiO_3_, Ti*_x_*P*_y_*, β-Ti, Ti_2_O, CaO, Ca_4_O(PO_4_)_2_, Ca_3_(PO_4_)_2_(TCP), and CaTi_4_(PO_4_)_6_ in Ti alloy/HA composites after high temperature sintering [24,25,26,27,28,29,30,31]. It reveals that there are complex reactions between Ti and HA when sintered at a high temperature. Although some new phases, such as CaO, TiO_2_, and CaTiO_3,_ were helpful to improve the biological properties of composite, the existence of reactions also decreased the mechanical properties of composite [26].

It has been found that novel biomaterials satisfying their comprehensive properties can be obtained through proper process by using different components. Carbon fiber and graphene have become common enhancement phases used in the composite due to their high thermo conductivity, low thermal expansion coefficient, high damping capacity, and excellent self-lubrication [3]. In fact, excellent self-lubrication can effectively improve wear resistance, which is also one of the main attractions for biomaterials. Moreover, the particle size of enhanced phases also affects the ductility, strength, and fracture properties of the composites, because nano-reinforcements can further enhance the performance of the composite due to the grain refinement strengthening. Besides, nanomaterials have higher an osteoblast adhesion rate and can promote the excretive function of osteoblast, which is beneficial to tissue growth in the next stage after transplantation [1]. Recently, carbon nanomaterials (graphene nanosheets and carbon nanotubes) have become promising reinforcement materials due to their high mechanical strengths and unique carbon structures [32]. For example, Hu et al. found that nano-indentation hardness values (11 GPa) of single-layer graphene oxide-reinforced Titanium matrix nanocomposite are almost three times of those of pure Titanium, and modulus value (200 GPa) is also significantly increased [33]. Addition of multi-layered graphene nanofiller in the Titanium matrix composite favored the improvement of mechanical properties, such as yield strength and wear resistance, which resulted from improvement of dislocation density through the Orowan mechanism [34]. Graphene in Titanium or Titanium alloys can stimulate biomineralization and promote the formation of mineralization necks, which can improve the biological activity of the composite [35]. Besides, cell interaction is affected by the state of additional graphene. Chetibi et al. found that the composite coating produced dense uniform nanoparticle morphologies after adding multi-walled carbon nanotubes (MWCNTs), which improved the adhesion strength between coating and substrate [36]. Moreover, other strengthening phases such as Zn element and some metal oxides can also improve the properties of materials due to their interactions with HA or Titanium alloys [37,38,39,40]. For example, Lugovskoy et al. found that the reaction between ion phosphate and Hydrated Titanium/Hydroxide from titania film can promote the formation of HA, which improved the interface bonding strength between HA and Titanium alloy substrate [16]. It was revealed that the HA/TiO_2_ composite coating containing Zinc element helps the osteoblast to grow with excellent bioactivity [37]. This was because the Zinc ion was released with decomposition of HA in the body fluids, except for the effect of the porous structure formed. Then, released Zinc ions produced solution-mediated effects on cytoactive. Besides, Zinc ions also absorbed proteins, which caused a series of cellular responses [37]. Nevertheless, there exist some problems of reunion in the nano-carbon phases, and integrity of sintered carbon nanophases may be destroyed by reactions with Ti [32,41]. Furthermore, specific effects and action mechanisms of the strengthening phases remain unclear [16,33,37,42].

Interfacial characteristics and microstructures are the key factors that can control the properties of either coating or uncoated composites. So far, research has been mainly conducted on preparation methods, microstructures, and mechanical properties, while their interfacial characteristics and strengthening mechanisms are not well defined. This article systematically summarizes the latest advances concerning the interface properties of Titanium alloy/HA composites, such as their interface reaction mechanisms, surface microstructures, etc. The strengthening mechanisms and performance evaluation of the Titanium alloy/HA composites are also analyzed. On this basis, the interface characteristics and mechanisms of the Titanium alloy/HA composites with enhanced phase are prospected. This provides a guideline for researching the Titanium alloy/HA composites.

## 2. Interfacial Characteristics of Titanium Alloy/HA Composites

### 2.1. The Mechanisms of Interfacial Bonding

**Figure 1:**
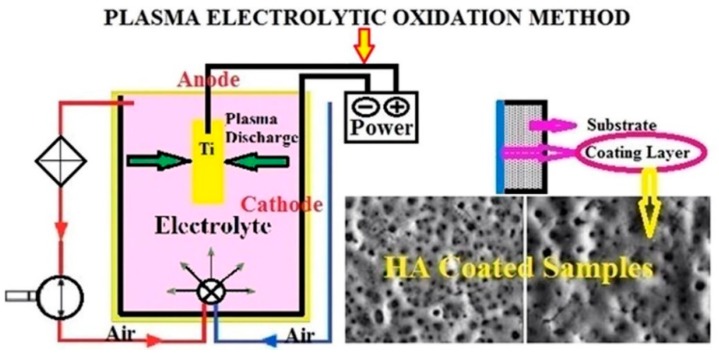
Morphology and schematic view of PEO method [15].

Recently, there have been two research methods that have been used on Titanium alloy/Hydroxyapatite composites: (1) Various coatings such as Hydroxyapatite, HA-Ti(C,N)-TiO_2_, HA-Ti, graphene-HA, etc. are coated on Titanium or Titanium alloy substrates using sol-gel method, laser cladding method, plasma spray method, plasma electrolytic oxidation, micro-arc oxidation method, and other methods [11,14,15,17,40,43,44,45]. Figure 1 shows the morphology and schematic view of Plasma electrolytic oxidation (PEO) method. (2) Titanium alloy powders, Hydroxyapatite, etc., are made into uncoated composites using powder metallurgy. In addition, the interface can be understood in the microstructural characterisation for the obtained layer or coating on a known substrate as a space between the matrix and reinforcing phases.

#### 2.1.1. Interfacial Chemical Reactions in Composite Coating

Some chemical reactions were identified in the interface of composite coating. Researchers [9,13,14,43,44] found that there are anatase (TiO_2_), rutile (TiO_2_), TiP_2_, Ti_4_P_3_, TCP (Ca_3_(PO_4_)_2_), CaTiO_3_, HA, Na_2_Ti_6_O_13_, etc., on the coatings on Titanium or Titanium alloy substrates prepared by plasma electrolytic oxidation [43] and plasma spraying [11]. These phases mainly resulted from the decomposition of HA and reactions between HA and Titanium alloy. Zhang et al. revealed that Na_2_Ti_6_O_13_ in the HA/TiO_2_ composite coating-deposited on the Ti6-Al-4V substrate is a product of reaction between TiO_2_ and sodium ions in the electrolyte (Equations (1)–(4)) [44]. Durdu et al. outlined complex reactions between Titanium alloy and HA in Equations (1) and (5)–(10) [45]. The coating processed by plasma electrolytic oxidation (90 min) had good adhesion strength with substrate due to chemical binding that resulted from the existence of calcium titanate [45]. The formation of TiO_2_ in the coating of HA/Ti composite was produced by the reaction between Titanium and oxygen in the air. The oxygen in the reaction resulted from reactions between Titanium and HA, though specific reactions were not clear [11]. Besides, the formation of calcium titanate was produced by the reactions of calcium oxide with Titanium or TiO_2_, and reaction between HA and Titanium [11].

(1) $$Ti-4e=Ti^{4+}$$

(2) $$4OH^{-}-4e=2H_{2}O+O_{2}↑$$

(3) $$Ti^{4+}+4OH^{-}=TiO_{2}+2H_{2}O$$

(4) $$2Na^{+}+6TiO_{2}+2OH^{-}=Na_{2}Ti_{6}O_{13}+H_{2}O$$

(5) $$Ti^{4+}+2OH^{-}↔TiO_{2}+2H^{+}$$

(6) $$3Ti^{4+}+4PO_{4}^{3-}↔TiP_{2}+2TiP+8O_{2}↑$$

(7) $$Ca^{2+}+OH^{-}↔CaO+H^{+}$$

(8) $$3Ca^{2+}+2PO_{4}^{3-}↔Ca_{3}(PO_{4}{)}_{2}$$

(9) $$10Ca^{2+}+6PO_{4}^{3-}+2H_{2}O↔Ca_{10}{\left(PO_{4}\right)}_{6}{\left(OH\right)}_{2}+2H^{+}$$

(10) $$Ca^{2+}+Ti^{4+}+3OH^{-}↔CaTiO_{3}+3H^{+}$$

It was found that Ti(C,N) and anatase are in the HA-Ti(C,N)-TiO_2_ composite coating [40]. The formation of Ti(C,N) was due to chemical reaction on the electrode surface and plasma-enhanced diffusion effect, and the formation of anatase was attributed to the interaction of plasma-enhanced diffusion and penetrant action of active oxygen atoms. The oxygen originated from water vapor, which was from electrolyte and decomposition reaction of HA [40]. In HA/Ti composite coating prepared by laser cladding, it was found that decomposition products of HA floated into upper layer [17], so that a clear interface between alloy area and coating would appear [17]. Besides, phosphorus resulting from decomposition reaction of HA reacted with Titanium and formed Titanium phosphorus compound, which could have been reserved in the alloy area, but calcium-phosphate compound floated into upper coating layer [17]. In graphene-HA composite coatings using electrochemical deposition method, it was found there is the existence of TiC produced by the reaction between graphene and Titanium, which resulted in the reaction bonding between Titanium and coating [46].

#### 2.1.2. Interfacial Chemical Reactions in Uncoated Composites

Compared with coating, there are similar complex chemical reactions in uncoated composite. Researchers revealed that some new phases, such as Ca_5_(PO_4_)_3_(OH), CaTiO_3_, Ti*_x_*P*_y_*, β-Ti, Ti_2_O, CaO, Ca_4_O(PO_4_)_2_, Ca_3_(PO_4_)_2_(TCP), CaTi_4_(PO_4_)_6_, etc., have appeared in Titanium alloys/HA composites prepared by powder metallurgy and other methods [24,25,26,27,28,29,30,31]. The presence of calcium titanate produced by the reaction between Titanium and HA showed the existence of interfacial chemical reactions [27]. The formation of β-Ti was due to the presence of Niobium, Tantalum, etc., which belong to β stable elements. In the high-temperature sintering process, Titanium element transformed from the original alpha-Ti to beta-Ti as sintering temperature increased. Then, the allotropic phase transition (β-Ti→α-Ti+β-Ti) occurred in the composite during furnace cooling, resulting in the formation of β-Ti. This was because that niobium and tantalum elements can reduce the temperature of allotropism transition of Titanium, expand the β phase zone, and increase the thermodynamic stability of β phase. Besides, the decrease of grain size increased the diffusion rate of Titanium in the sintering process and further promoted the phase transition. Nath et al. revealed that TCP in the Ti/HA composite contain β-TCP or α-TCP after sintering, which depends on cooling rate in the sintering process. Rapid cooling resulted in the existence of more alpha-TCP, while conversely slowed cooling rate caused improvement of content of β-TCP [47]. Research mentioned that sintering processes of Ti-10% HA composite at 1200 °C is illustrated as Equations (11)–(14) [1]. As far as Titanium oxide was concerned, the interdiffusion easily occurred between oxygen and Titanium elements in the HA/Ti composite [1]. Oxygen element diffused into lattice of Titanium until saturation, resulting in the formation of Ti_2_O. The diffusion rate gradually decreased with the formation of Titanium oxide. Afterward, Phosphorus and Calcium also diffused, causing the changes of the Ca/P ratio and the properties of composites [1]. The formation of Titanium phosphide was shown in the Equation (15), which resulted from the reaction of Equations (16)–(18) [23]. Besides, Kumar et al. revealed that the existence of CaTi_4_(PO_4_)_6_ is caused by Equations (19)–(20) [31].

(11) $$Ca_{10}{\left(PO_{4}\right)}_{6}{\left(OH\right)}_{2}\to Ca_{10}{\left(PO_{4}\right)}_{6}{\left(OH\right)}_{2-2x}O_{x}+xH_{2}O$$

(12) $$Ti+2H_{2}O(g)\to TiO_{2}+H_{2}$$

(13) $$Ca_{10}{\left(PO_{4}\right)}_{6}{\left(OH\right)}_{2}+2TiO_{2}\to 3Ca_{3}(PO_{4}{)}_{2}+CaTi_{2}O_{5}+H_{2}O$$

(14) $$Ca_{10}{\left(PO_{4}\right)}_{6}{\left(OH\right)}_{2}\to Ca_{4}P_{2}O_{9}+2Ca_{3}(PO_{4}{)}_{2}+H_{2}O$$

(15) $$5Ti+3P\to Ti_{5}P_{3}$$

(16) $$Ca_{10}{\left(PO_{4}\right)}_{6}{\left(OH\right)}_{2}+TiO_{2}\to Ca_{3}(PO_{4}{)}_{2}+CaTiO_{3}+H_{2}+1/2O_{2}$$

(17) $$Ca_{3}(PO_{4}{)}_{2}+1/2O_{2}+\mathrm{Zr}\to CaZrO_{3}+2P$$

(18) $$Ca_{3}(PO_{4}{)}_{2}+1/2O_{2}+3\mathrm{Ti}\to 3CaTiO_{3}+2P$$

(19) $$Ca_{10}{\left(PO_{4}\right)}_{6}{\left(OH\right)}_{2}\to 3Ca_{3}(PO_{4}{)}_{2}+CaO+H_{2}O↑$$

(20) $$3Ca_{3}(PO_{4}{)}_{2}+4TiO_{2}\to CaTi_{4}{\left(PO_{4}\right)}_{6}+8CaO$$

The existence of Ti_2_Ni, Ni_3_Ti, and Ni_4_Ti_3_ in uncoated composite not only increased brittleness and reduced biological compatibility but also caused cavitation [6]. There were NiTi and other secondary phases (Ti_2_Ni and Ni_3_Ti) in NiTi-HA composite [6]. Because the electric field effect in the process of spark plasma sintering greatly enhanced atomic diffusivity. Moreover, large current pulse caused the rapid formation of joule heat. Thus, Titanium and Nickel realized full alloying and formed Nickel-Titanium compounds [6]. As far as Titanium/HA composite is concerned, it is of great significance to reduce reaction degree and maintain the enhanced effect of the second phases that were added. Dehydroxylation only occurred in the stable HA above 1200 °C, but the addition of Titanium made dehydroxylation occur about 1200 °C [48]; then, HA continued to decompose or react with Titanium [48]. However, Wang et al. prepared rod-like Titanium-doped Hydroxyapatite nanopowder using chemical co-precipitation and microwave technology [49]. The result did not show any new phases detected in the composite, but diffraction peaks of HA shifted [49]. This indicated that the addition of Titanium inhibited decomposition of HA and thermal reactions with Ti at high temperature. The thermostability of HA was thus improved. Since Titanium atoms entered the areas between HA crystals and even replaced the Phosphorus atoms, the crystal structure of HA could be changed [49]. Besides, the study by Chang et al. showed that HA-5% (Ti-33 wt. % Fe) composite consisted of HA and some new phases, such as Ti, Fe, TiFe, β-CaTiO_3,_ etc. [12,38]. The contents of β-TCP and Ca_4_O(PO_4_)_2_(TTCP) were significantly decreased, as these second phases were not detected by XRD [12,38]. This showed that the addition of Iron can inhibit decomposition of HA and chemical reactions with Ti, which are related to faster diffusion rate of Iron element in Titanium at 836 °C [12,38].

#### 2.1.3. Interfacial Mechanical Effect

In addition to the aforementioned chemical interactions, there are a variety of combinations of physical interactions in materials [17,50]. For example, Akmal et al. found that nHA/NiTi composite is transformed into lamellar substance from spherical and lamelliform material after ball milling. This was because cold processing in the process of milling can cause grain refinement and cold welding [51]. Arifin et al. revealed that as the temperature rises, atomic diffusion rate is improved in HA/Ti-6Al-4V composite, promoting the combination of the composites [28]. Besides, Dom et al. found that superplastic deformation of surface roughness of the substrate and small grain size also promotes interface bonding between coating and substrate [52]. On the other hand, Chang et al. studied interfacial reactions in Ti-Fe particles reinforced HA composite and found that the addition of Iron can restrain reactions of HA, which can make pure Titanium retained in the matrix [53]. There was a good combination in the interface of composite, although some pores were found. However, a few pores produced by interaction between HA and Titanium were found to be favorable to the suitable interfacial bonding [53].

**Figure 2:**
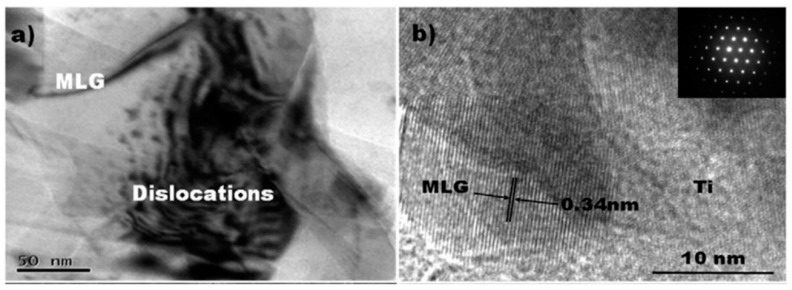
(**a**) TEM image showing dislocations within Titanium phase; (**b**) HRTEM image showing clean MLG-Ti interface (inset the selected area diffraction patterns of Titanium) of 0.5 wt. % MLG/Ti composite [34].

Based on the observation of high-resolution transmission electron microscopy (HRTEM) image of Titanium-matrix composite containing multilayered graphene nanomaterials (Figure 2b), no intermediate phases were found, which showed that multi-layer graphene (MLG) was combined with matrix by mechanical effect [34]. Similarly, no clear evidence was found to verify existence of chemical reactions at the interface of HA/graphene composites [54]. Combining by van der Waals bonding was probably the most likely way, which resulted from the complex crystal structure of HA and folded surface morphology of graphene [54]. Zakharov et al. studied interaction between graphene and HA, and found that HA/GO (graphene oxide) has lower thermostability compared with HA/CNT (carbon nanotube) [55]. Interaction between graphene and HA was based on the agglomeration of active OH groups in the HA nanocrystals formed on the surface and edges of graphene, causing the decrease of HA nanocrystal size and the increase of solubility [55]. Besides, some researchers have shown that large increase of fracture properties is due to intense interfacial bonding from mechanical occlusion between graphene and matrix [54].

### 2.2. Microstructure and Formation Mechanism

It is well known that microstructure of materials plays an important role in mechanical properties and biological activity. Since uniform distribution of reinforced phases generally favors the improvement of performance, the materials with uniform distribution of fillers have been made in different methods [5]. Yang et al. prepared continuous, uniform, and densified HA/Ti composite coating, using adequate melting in the process of laser cladding process [17]. Titanium particles completely melt in the process of preparation due to high heat released by laser, causing the formation of Marangoni convection and molten pool with a high temperature gradient [17].Therefore, the coating rapidly cooled and formed densified material after moving of laser beam. Singh et al. found that after heat treatment, the elements were well distrusted on surface of HA-10Al_2_O_3_ and HA-10ZrO_2_ coatings, because amorphous phase is easy to recrystallize in the process of heat treatment [39]. For enhancement phases, it was revealed that the addition of CNT, TiO_2,_ and GNFs (graphene nano flakes) results in the formation of uniform density structure in materials [56,57]. TiO_2_ (9.0 × 10^−6^ °C^−1^) reduced differences in thermal expansion coefficient between HA layer (15.2 × 10^−6^ °C^−1^) and Titanium substrate (Ti, 8.6 × 10^−6^ °C^−1^) [56]. Besides, TiO_2_ particles can supplement pores between CNT and HA, and improve adhesion strength between coating and substrate, which are beneficial to the formation of interface containing less microscopic cracks [57]. CNT also played the role of inhibiting crack propagation due to the effect of transferring residual stress, which promoted the formation of uniform and densified structure [57]. However, it was found that partial agglomeration occurs when the content of additional CNT is too high, which can decrease strengthening effect of CNT [58]. On the other hand, it was found that the (300) crystal plane of HA is naturally parallel to graphene surface with consistent interface binding.Moreover, graphene appears as an amphiphilic macromolecule with hydrophobic regions embedded in hydrophilic zones,which is beneficial to the uniformdispersionof HA particles [59].

**Figure 3:**
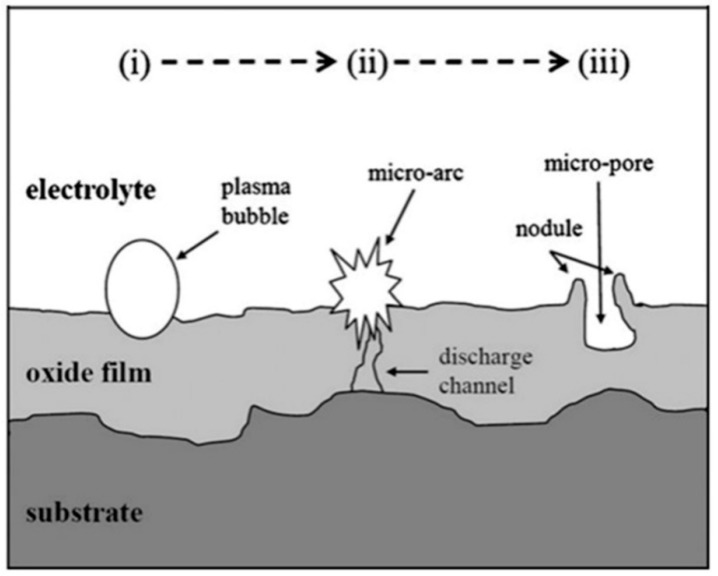
Schematic diagram of the PEO coating to form the oxide film by plasmabubble implosion: (i) Plasma bubbles take place on surface; (ii) Micro-arcs, whichare generated through the narrow discharge channels, lead to the implosion; and (iii) This implosion makes micro-pores in the oxide film [61].

In fact, compact density value of materials generally does not reach 100%, and pores can influence performance of materials. However, the size and number of pores can be controlled to a certain extent [9,40,60]. Researchers studied HA, HA-TiO_2_ coating prepared by plasma electrolytic oxidation on Titanium alloy substrate and found that porous morphology on the surface of HA, HA-TiO_2_ coating was formed in three steps [61], as shown in Figure 3: (1) The voltage in the electrolyte increased and negatively charged ions rapidly moved around surface of anode substrate after experiment, which caused the formation of bubbles; (2) As time increased, the plasma rapidly expanded, and the bubbles around the substrate collapsed; (3) The collapse of these bubbles caused the formation of miniature volcano-shaped pits and pores on the surface of coating. Diameter of pores changed with difference of applied voltage [15]. There are curing droplets, pores, and micro-cracks on the surface of HA-TiO_2_ coating after plasma spraying and post-heat treatment [62]. The micro-cracks after plasma spraying resulted from rapid quenching in the preparation process [62]. Besides, unmolten particles resulting from improper sintering process were found on the surface of coating [62].

**Figure 4:**
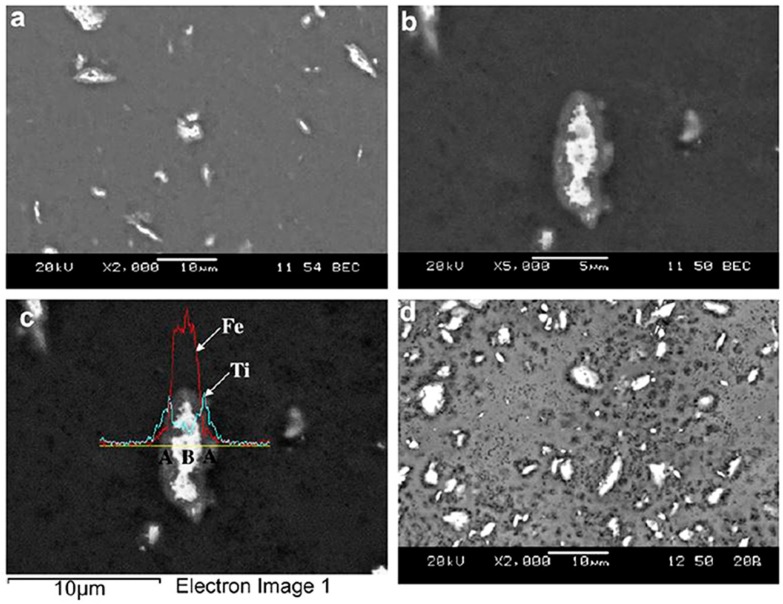
Backscattered SEM images and EDS analysis of HA-Ti-33 wt. % Fe composites sintered in vacuum at 1000 °C; (**a**) image of HA/5% (Ti-33 wt. % Fe); (**b**) high-magnification image; (**c**) EDS line scan across a typical Ti-Fe particle and (**d**) image of HA/15% (Ti-33 wt. % Fe) [63].

Moreover, there are some other special features in Titanium alloy/HA composites. For example, researcher [63] found that Ti-33 wt. % Fe particles in HA/Ti composites containing Iron show a spherical shell structure and were uniformly distributed in the HA matrix, as shown in Figure 4. The outer shell was Titanium and the inner was Iron. This interface topography was formed in three steps [38]: (1) Ti and Fe elements were randomly distributed when Ti-33 wt. % Fe composite particles were added. As the temperature rose, Ti reacted with water produced by a decomposition reaction of HA and formed Titanium oxide, which further reacted with HA. The driving force generated by these reactions made Titanium move rapidly and formed a shell composed of Titanium elements between HA matrix and Ti-33 wt. % Fe particles; (2) The Iron closely related to HA diffused into apatite crystals. Fe has a faster diffusion rate in Titanium at 836 °C, promoting the densification of composites and growth of Titanium shell. Diffusion process of 2h caused the densification of composites, growth of Titanium shell, and good interfacial bonding; (3) The diffusion process of Iron into Titanium was inhibited when Iron reacted with Titanium and formed TiFe. Residual Iron was retained between Ti-33 wt. % Fe particles and formed an Iron-based core [38]. Marcu et al. found that there are some black zonal materials and columnar grains in Ti-6Al-7Nb-HA composite prepared by selective laser melting [22], because the microstructure of composites was affected by local heating in the preparation process. In a study of the influence of electrolyte on the surface of pure Titanium in the process of plasma electrolytic oxidation, it was found that the oxide films coated in an electrolyte containing Potassium Pyrophosphate easily form biomimetic apatite in simulated body fluid, because higher electrochemical resistance and heat generated in the preparation process could produce crater-like structural micropores, resulting in high surface roughness [61].

## 3. Strengthening and Toughening Mechanisms of Titanium Alloy/HA Composites

### 3.1. Interfacial Bonding Strength between Coating and Substrate

The coating can be prepared by plasma spray deposition, hot isostatic pressing, thermal spraying, immersion plating, pulsed laser deposition (PLD), electrophoretic deposition (EPD), sol-gel and ion beam assisted deposition (IBAD), etc. There exist some problems in the coating materials, such as high porosity, uneven thickness distribution, low crystallinity, and weak bonding strength [18]. In fact, there are some factors that can affect bonding strength, such as mechanical anchorage, metallurgical process, chemical reaction, and residual stress [18].

#### 3.1.1. Physical Enhancement

**Figure 5:**
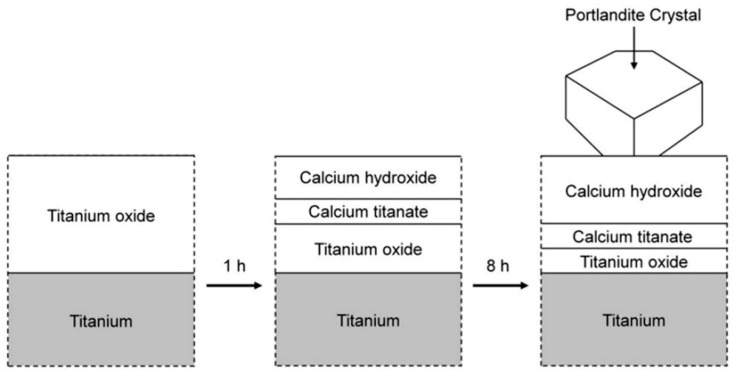
Schematic cross-sectional illustration of the modification of the outermost surfaces of Titanium specimens as a function of pretreatment time in the hydrothermal liquid [64].

It has been revealed that specific shape and uniform structure can improve bonding strength of coating [15]. Dinçer et al. found that hydrothermal pretreatment improves the bond strength between HA coating and Titanium alloy substrate [64], because after hydrothermal pretreatment, the nucleation and formation of Calcium Hydroxide improved surface roughness, as shown in Figure 5 [64]. The surface of transplant material tightly combined with mineralized bone, due to the mechanical lock interaction when surface roughness is less than 10 μm [64]. During heat treatment, volume shrinkage occurred around the regions of phase inversion and recrystallization, resulting in the decrease of bonding strength between coating and substrate [39]. Besides, it was found that after heat treatment, the decreased bonding strength concerning HA composite coating is related to the diffusion of Oxygen in Titanium alloy substrate [39]. When Oxygen element diffused into the surface of substrate, a hard and brittle oxygen-rich layer was formed, thereby reducing the interface bonding strength between substrate and coating. In fact, the interfacial bonding strength between coating and substrate was controlled by the size of cohesive strength. However, difference in thermal expansion coefficient and diffusion of Oxygen greatly reduced the cohesive strength at the interface after heat treatment, which in turn affected the interfacial bonding strength between the coating and the substrate [39].

Moreover, the addition of graphene [46,59] and carbon nanotubes [56,57,58,65] effectively increased bonding strength between composite coating and Titanium substrate, based on the following facts: (1) The introduction of graphene resulted in the formation of nanoscale apatite crystals and increased contact areas between coating and substrate; (2) The presence of graphene reduced the difference of thermal expansion coefficient between Titanium alloy and coating; (3) Graphene can form an effective reinforcement network through bridging due to its excellent mechanical properties, which further enhanced load transfer of composites [46]; (4) CNTs can also formed an enhanced network and prevented coating stripping [56]. Besides, the addition of TiO_2_ layers at the interface between coating and substrate promoted the bonding between HA layer and Titanium substrate, because the TiO_2_ layers decreased the differences in thermal expansion coefficients and reduced residual stress in the coating [56]. Similarly, it was found that the Zirconia intermediate layers also increased bonding strength between HA coating and substrate, due to the mechanical lock effect and element diffusion mechanism [39]. Moreover, Zirconia intermediate layers can reduce the mismatch of thermal expansion coefficient between HA and substrate [39].

#### 3.1.2. Chemical Enhancement

**Scheme 1:**
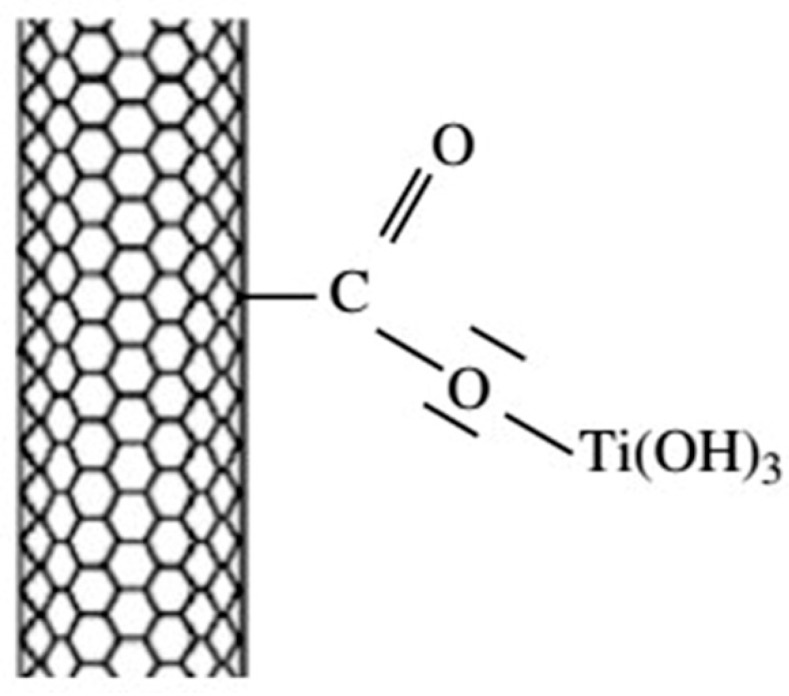
Reaction of MWCNTs with TiO_2_ NFs (nanofibers) [36].

**Figure 6:**
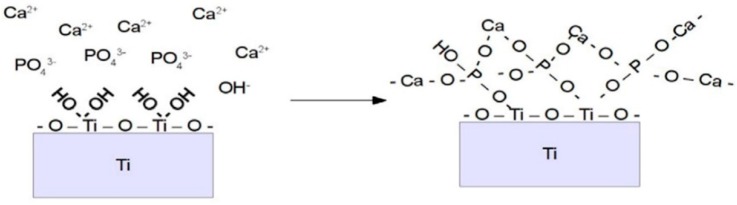
Schematic view of bonding Hydroxyapatite on the Titanium surface [16].

Studies showed that reactions between Titanium alloy substrate and HA coating can occur to enhance interfacial bonding strength [13,19]. It was revealed that calcium titanate or Titanium-phosphorus compounds are formed due to reactions between Titanium and HA coating, which results in the appearance of chemical bonding between interfaces and enhances interfacial bonding strength [19]. CNTs were added as reinforcements to Ti or HA, while the addition was not always successful at enhancing interfacial bonding strength [66,67]. In a study of the structure of HA/MWCNTs/TiO_2_/Ti, MWCNTs between HA and TiO_2_ layers effectively improved bonding strength [36]. In the experiment, ammonium salt of carboxyl carbon nanotubes (MWCNT-COONH_4_) could produce chemical compatibility with the surface of TiO_2_. Surface functional groups (O=C–O or COO–) provided active sites for orientation reactions between anatase nanocrystals and MWCNTs. The specific reactions are shown as Equations (21) and (22) and Scheme 1 [36]. In addition, it could form a Titanium oxide layer on the surface of Titanium or Titanium alloys by micro-arc oxidation [68], plasma electrolytic oxidation [16], or Titanium surface anodization [19]. The reaction between formed Hydrated Hydroxide Titanium and Phosphate ions further promoted the formation of HA (Figure 6) and improved interfacial bonding strength [16]. Moreover, the obtained porous Titanium Dioxide layer was mostly rutile, and the O–O bonds of Titanium Dioxide layer was highly compatible with the Ca–Ca bond in apatite [19].

(21) $$TiO_{2}+2H_{2}O+\overset{K}{⟷}Ti{\left(OH\right)}_{4}$$

(22) $$Ti{\left(OH\right)}_{4}\overset{K}{⟷}{\left[Ti{\left(OH\right)}_{3}\right]}^{+}+OH^{-}$$

### 3.2. Strengthening and Toughening Mechanisms of Composites

In recent years, researchers have prepared uncoated composites by some methods such as powder metallurgy, avoiding interface problems of coated materials. It is of great significance to study the strengthening and toughening mechanism of composites.

#### 3.2.1. Grain Refinement Strengthening

There are some benefits to use nanoscale phases in Titanium alloys/HA composites. It is well known that nano-sized particles have good sintering rate due to their short migration distance and high driving force. In addition, nanoparticles caused uniform distribution of reinforcing phases, resulting in high density and uniform structure [51]. Therefore, mechanical ball milling was frequently used to obtain smaller grains of composites to obtain higher hardness and density before the subsequent sintering process [21,29,69,70], because agglomerated particles were gradually refined due to continuous cold welding and fracture process in the high-energy ball milling. Particle shape was also changed from needle-like or disk-like to spherical. Thus, the hardness and corrosion resistance of composites increased with the improvement of milling time due to the grain refinement strengthening effect [24]. However, the refined particles continued to agglomerate, and the mechanical properties reduced if the ball milling time was too long [32].

**Figure 7:**
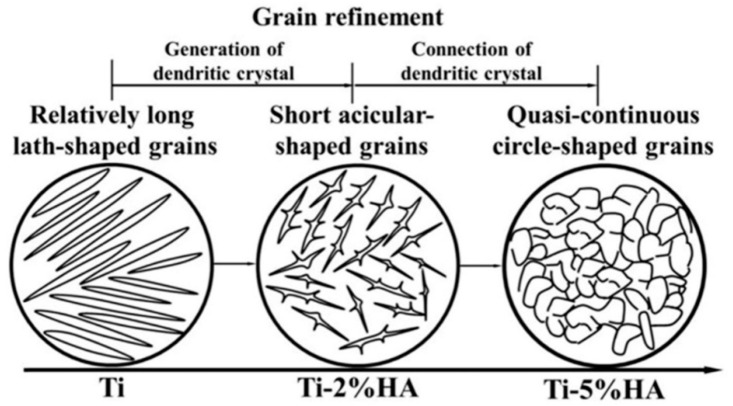
Schematic diagram of the microstructure evolution of SLM-processed Ti-nHA composites with different nHA addition [4].

**Figure 8:**
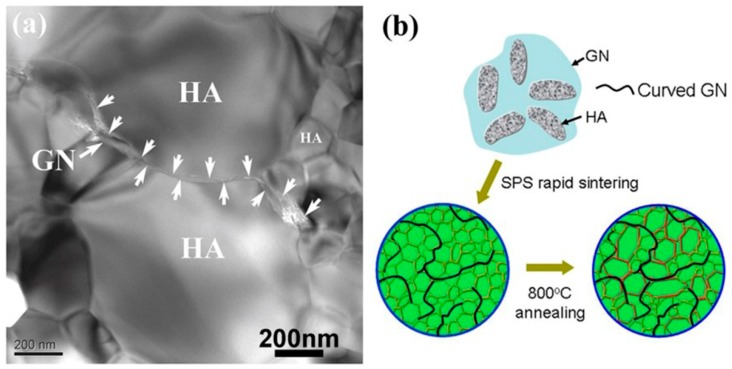
Restrained HA grain growth by GN(graphene nanosheet) and abnormal growth of the grain along the direction parallel to GN; (**a**) TEM image of the HA-GN composites showing that GN is predominantly located at the HA grain boundaries, forming a serial wall zones isolating individual HA grains, and abnormal HA grain growth is seen along the direction parallel to GN; and (**b**) schematic depiction of the composites illustrating evolvement of the HA grains during the SPS processing and following heat treatment [54].

On the other hand, the addition of some second phases may also produce the refinement effect. For example, HA composites doped with Titanium have smaller particles than those with pure HA, as Titanium inhibited the growth of particles during sintering [49]. When the content of Titanium was 0.8%, the composite exhibited nanoscale rod-like crystals [49]. It was found that as the content of the added HA increased, Titanium particles changed from slat particles to short needle-like or quasi-continuous annular particles, as shown in Figure 7 [4]. The analysis showed that grain refinement is achieved through molten pool dynamics and polymorphic nuclear effects [4]. Besides, graphene also plays a part in grain refinement strengthening [42,54]. Because the graphene nanosheets were present in the HA after high temperature heat treatment, the nucleation and growth of HA particles during preparation processing were along the direction of graphene sheets. Therefore, grain growth of HA was effectively suppressed due to the segregation between the HA particles by the graphene, as shown in Figure 8 [54]. Moreover, sintering temperature also played an important role [42]. It was found that Titanium-graphene composites had larger particles than pure Titanium, which was due to the use of nano-sized Titanium particles and micron-scale graphene sheets [71].

#### 3.2.2. Second Phase Strengthening

**Figure 9:**
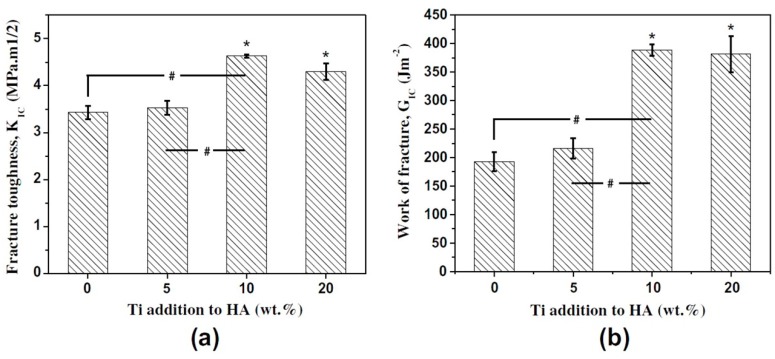
(**a**) Variation in fracture toughness and (**b**) work of fracture with wt. % Ti in HA. Data in (**a**) and (**b**) have been represented as mean ± standard error. Dunnett’s *t*-test (two-sided) was used to compare the HA with HA-Ti samples (marked with *), while Dunnett’s C-test was used to find the most significant difference among all samples (marked with #). Statistical analysis shows the most significant difference at 0.05 level for *n* = 5 [31].

It has been found that after adding HA, the hardness of Titanium alloy/HA composites increases, while the sintering capacity decreases [24]. This was because HA has higher hardness value and biological activity, but higher porosity in the reactions led to a decrease in sintering capacity [24]. Akmal et al. revealed that the addition of HA to NiTi matrix increases dislocation density of composites, providing more sites of nucleation and precipitation [51]. Moreover, Gibbs’ free energy was reduced, and new phases were formed as stable precipitates. When HA content was 6%, hardness value of composite reached the maximum due to the presence of tough phases, such as Ni_3_Ti. However, the presence of these tough phases increased brittleness of composite, resulting in cavitation and decrease of biological activity [12]. Conversely, Titanium can increase toughness of HA/Ti composites [12,31,72], as shown in Figure 9 [31]. This was due to the bridging effect of ductile metals [73]. It was revealed that 5 wt. % Ti-Fe particles can enhance fracture toughness, bending strength, fatigue life, and other mechanical properties concerning HA-(5% Ti-Fe) composites [63], since Fe can inhibit the reactions of HA and Ti in HA-(5% Ti-Fe) composites. The remaining tough Titanium phases consumed more energy during fracture process due to plastic stretching effect, and thus mechanical properties were improved. Therefore, adjusting the composition of composites can enhance fracture toughness due to mixed shielding mechanism [74]. Besides, nano-carbon materials can enhance mechanical properties [32,75,76,77]. Hu et al. revealed that Vickers hardness of Titanium-graphene composites initially increases with increasing of graphene content [71], as graphene itself has excellent mechanical properties that can carry loads and block dislocation movements in the composites. When the content of graphene was high, Vickers hardness of Titanium-graphene composites decreased [71], as graphene is a kind of 2D material, and its binding force with the matrix is weak. When the content was too high, graphene tended to agglomerate to form pores in the composites, reducing the performance of composite [71]. Yang et al. experimentally found that when the content of graphene is 0.4 wt. %, the aspect ratio of graphene decreases due to the agglomeration of graphene at the pores, resulting in a slight decrease of compressive strength of composite [42].

#### 3.2.3. Solution Strengthening

From XRD analysis, it was found that the strength of α-Ti in the porous Ti/HA composites decreases after sintering, indicating that the oxygen in the HA in the sintering process may diffuse into Titanium matrix in a role of solution strengthening [78]. However, diffusion of oxygen caused brittle fracture at the sintering necks of Titanium if the porosity was high [78]. In the (Ti-13Nb-13Zr)-10HA composites prepared using SPS at different temperature, it was found that the shift of Titanium peak in the XRD is due to changes in crystal parameters because of solid solution and sintering defects. Phosphorus and oxygen atoms with smaller radius and lower activation energy diffused into Titanium substrate. In addition, Niobium, as a β-stabilizing element, incorporated into Titanium to form a solid solution, except for the fact that it promoted phase transformation of Titanium [24]. In fact, as ball milling time increased, the Nb element peaks gradually disappeared in the XRD of Ti-35Nb-2.5Sn/10HA composites, indicating the solid solution of Nb [69]. It was found that the formation of supersaturated solid solution of Ti(Nb) and Ti(Zr) in (Ti-13Nb-13Zr)-10HA composites improved properties of composites by solid solution strengthening, although crystal parameters of Titanium are changed [79]. Studies showed that Sn element also formed Ti(Nb, Sn) solid solution resulting in solution strengthening [69]. It was revealed that as the stannum content increases, stannum diffuses into the crystal lattice of Titanium to form solid solution in Ti-35Nb-xSn/15HA composites. In addition, Stannum, as a α-stabilizing element, stabilized α-Ti phase and inhibited phase transitions [80].

#### 3.2.4. Cracks and Pulling Out Mechanism

**Figure 10:**
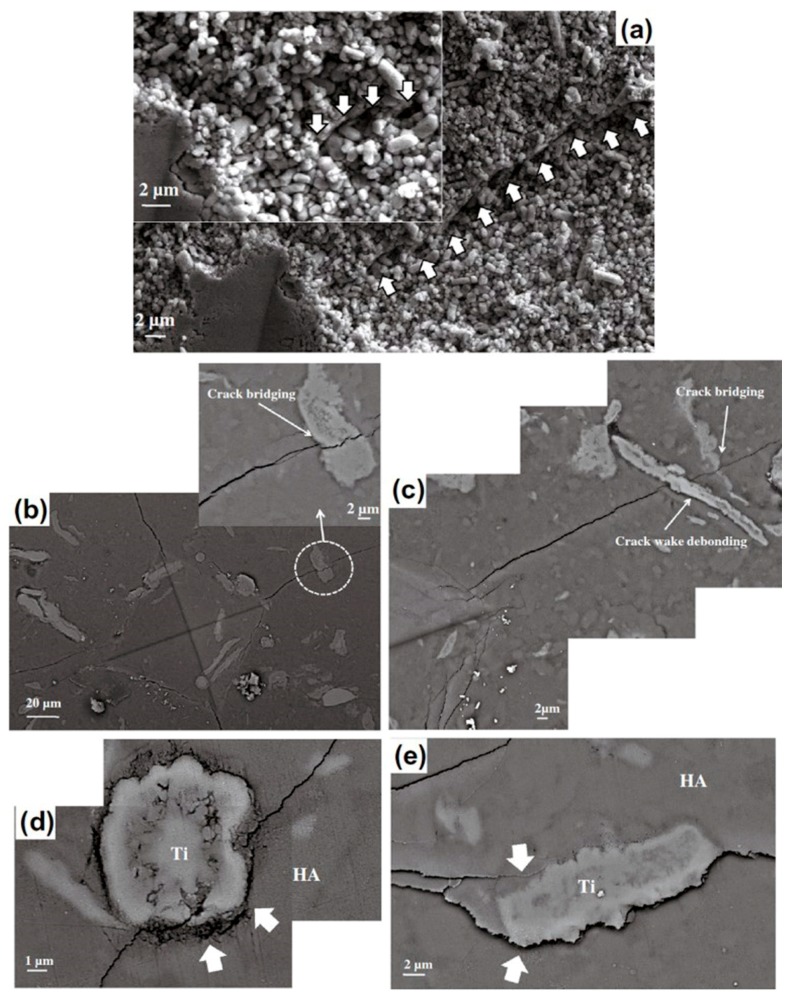
Representative secondary electron SEM micrographs of chemically etched HA (**a**), showing the indent (500 g load) region and crack path (marked with a white arrow); (**b**,**c**) represent the backscattered electron SEM images of HA-10 Ti, showing the indent (at 1000 g load) region and crack path. The crack bridging (marked on (**b**,**c**)) by lath-shaped/elongated Ti particles and crack wake debonding (marked on (**c**)) are visible. The crack wake debonding at the HA/Ti interface with spherical and lath-shaped particles is clearly visible and marked with white arrow on (**d**,**e**), respectively [31].

Micro-cracks that appear in composites contribute to the performances enhancement. It was found that the increase of fracture toughness and bending strength in the prepared HA-5 wt. % Ti-Fe composites is related to the Ti-Fe reinforcement particles showing the effect of plastic tensile and extended crack bridging [12,81]. In addition, toughening mechanisms caused by crack bridging, branching, and deflection were found in the Iron-containing HA/Ti composites, which increased crack propagation resistance and improved mechanical properties [12,81]. Additional energy of crack tip distributed in the fragile second-phase solid was found to dissipate in the shear experiment. Therefore, this reduced the driving force for crack propagation, enhanced the ability of resisting crack propagation, and thus increased fracture toughness of Ti-6Al-4V/HA composites [74]. Besides, crack propagation distributed in a ductile second phase, such as Titanium, was hindered by forcing cracks to pass or change direction, and thus fracture toughness of material was enhanced [20]. In addition, high fracture toughness (about 4.7 MPa m^1/2^) of HA-10Ti composites was attributed to the crack wake peeling and crack bridging at the interface [31,82], as shown in Figure 10 [31].

Fiber bridging and pull-out mechanisms are important factors in composites. In MWCNTs/HA reinforced composite with 3 wt. % MWCNTs, it was found that fracture toughness and bending strength of composite increased by 260% and 50%, respectively, compared with HA composite [83]. Since CNTs were evenly distributed in HA matrix and were tightly connected with HA; the pull-out mechanism of CNTs enabled composites to withstand loads and prevent crack propagation. In addition, the CNTs played the roles of bridging and interfacial bonding between HA and CNTs, facilitating load transfer and improving mechanical properties. It was found that as the content of MWCNTs increases, mechanical properties are reduced due to agglomeration of MWCNTs [84]. It was evident that some graphenes of about 2–4 μm are pulled out on the fracture surface of HA/GNFs composites with even distribution in HA matrix, demonstrating the graphene pull-out toughening mechanism [54].

#### 3.2.5. Integrated Mechanisms

Various experiments have demonstrated that the increased density of composites caused by heat treatment, sintering process, or uniform distribution of elements can improve mechanical properties of composites [20,31,63,85,86]. The compressive strength of Ti-40Zr-10Cu-36Pd-14HA composites gradually was found to decrease because of the following facts [87]: (1) Low density formed during sintering; (2) reduction of contact areas of vitreous alloy powder during sintering, decreasing the number of sintered necks formed between the powder particles; (3) partial segregation of glass-phase alloy powders. In HA/(Ti-33 wt. % Fe) composites prepared by pressureless sintering, HA, Ti, Fe, and some secondary phases (TiFe, CaCO_3_) were identified, and with the increase in Ti-33 wt. % Fe particles, TCP and TTCP appeared. This showed that there is some decomposition and chemical reactions between HA and Ti, and thus secondary phases are formed. These reactions affected the compactness and integrity of composites and reduced the mechanical properties [88].

Research showed that a large number of pores in the Titanium matrix weakened interfacial bonding at 950 °C [63].Thus, the increase of mechanical properties was limited, compared with pure HA. The increase of porosity at the interface weakened the interfacial bonding of composites at 1050 °C, and thus some interfaces were exfoliated when the crack encountered Ti-Fe particles, which made plastic stretch areas increase; more fracture energy was absorbed, and mechanical properties of composites were improved [63]. Besides, study showed that a small amount of pores at the boundary in Ti-Fe particle-reinforced HA composites can promoted interfacial bonding between the two components due to the interaction of HA and Titanium [53].The enhancement mechanisms of single-layer graphene oxide-enhanced Titanium-based nanocomposites included dislocation strengthening as well. For example, residual compressive stress was identified in multilayer graphene, while residual tensile stress in the Titanium matrix was due to the difference of thermal expansion coefficient [34]. Moreover, the residual stress at the interface was high enough to generate dislocations, and high-density dislocations released matrix residual stress for the enhancement. Moreover, a large number of dislocations were found in Titanium particles from the TEM results (Figure 2a) [34].

## 4. Performance Evaluation of Titanium Alloy/HA Composites

### 4.1. Fracture Behavior Evaluation of TitaniumAlloy/HA Composites

**Figure 11:**
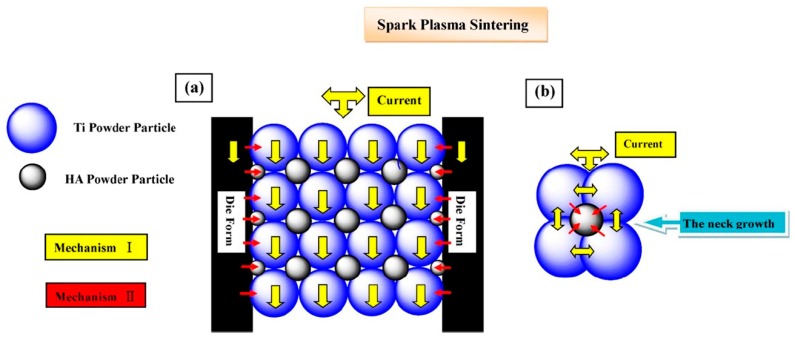
Schematic illustration of the local current distribution and the neck growth between particles of Ti-HA biocomposites. The yellow arrows indicate the effect of mechanism I (Spark plasma discharge mechanism), and the red arrows indicate the effect of mechanism II (Heat-transfer mechanism). (**a**) Since the electrical conductivity of HA was much lower than Ti, the current preferentially flowed to Ti particles and the die body; (**b**) High temperature plasma (spark plasma) generated in the gaps between Ti powder particles through Mechanism I, contributing to vaporization and themelting of the particle-contacting zones. Then, lots of joule heating of HA particles got from the surrounding Ti particles and die form through Mechanism II [30].

**Figure 12:**
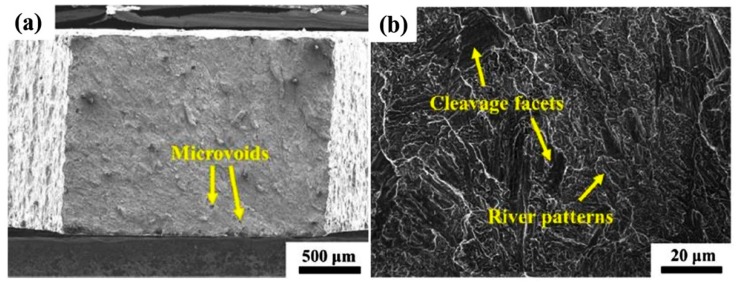
Fracture surface of the tensile samples (**a**,**b**) overview and magnification image of Ti-2%HA sample [4].

The fracture behavior and mechanism are critical factors for studying failure of composites. Balbinotti et al. found that the HA in the Ti/HA composite decomposed into Calcium Titanate, TCP phase, and Ti*_x_*P*_y_* at about 1026 °C. If these phases were formed at the boundary of Titanium particles, the composites exhibited grain boundary fracture [89]. Sintering mechanism of SPS is a combination of heat transfer mechanism and spark plasma discharge mechanism for Ti-HA composites, as shown in Figure 11 [30]. Therefore, secondary phases with a large difference in thermal expansion coefficient and electrical conductivity caused deterioration of sintering effect, resulting in the appearance of obvious macro cracks in Ti-30% HA composites [30]. Cleavage steps and river patterns were observed on the compressive fracture surface of Ti-5/10% HA composites, and local cleavage fracture was caused by weakened bending between thin pore walls. At first, microscopic cracks appeared at the edges between microscopic pores. Then, cracks expanded as the pressure of compression process increased. Next, macroscopic cracks formed when the pressure reached a certain level [30]. It was found that the stress-strain curves of NiTi porous composites prepared by SPS exhibited early failure with different degrees [6]. Due to characteristics of SPS method, sintering process can be achieved in a very short period of time, forming high porosity and interconnected pores. Thus, microscopic cracks propagated along pore walls. During the compression experiment, deformations around micro-cracks weaken and fracture inter-connected pore walls, producing macroscopic cracks. Besides, HA is a hard and brittle phase, and its plasticity is low. Therefore, connected holes broke and the stress rapidly decreased when the stress reached an approximate linear elastic deformation stage [6]. Some researchers have observed cleavage cross sections and river patterns on the fracture surface of Ti-2%HA composites [4], as shown in Figure 12. Rapid cooling in the preparation process supersaturated the phosphorus and oxygen elements in solid solution. As the content of phosphorus and oxygen elements increased, new phases such as Ti_5_P_3_ and TiO*_x_* appeared at the boundary of composites. Afterward, the cracks expanded rapidly due to the existence of these hard and brittle phases, resulting in the fracture of composite [4].

### 4.2. Biological Evaluation of Titanium Alloy/HA Composites

The biocompatibility of composites is crucial when they are transplanted into the human body. Moreover, HA is regarded as the most commonly used biomaterial because of its bone-like composition and excellent biological properties. Some researchers also revealed that HA-containing materials prepared by different techniques have excellent biocompatibility [6,11,73,90]. Wang et al. revealed that the cell activity on the surface of Ti-35Nb-2.5Sn/15HA composites is 1.4 times than that of pure Titanium [80]. It was revealed that when Titanium alloy/HA materials composites are immersed in simulated body fluids, Ti-OH and other functional groups firstly react with ions in simulated body fluids to form CaTiO_3_ and other complexes which are considered as the sites of nucleation and growth of apatite layer [79]. Besides, the increase of sintering temperature also promoted diffusion of ions, which in turn enhanced biological activity of materials [79]. Anawat et al. revealed that formation of HA layer on the surface of Ti-HA composites may be due to the formation calcium hydroxide resulting from the hydration of fine calcium oxide particles [91].In addition, researchers discovered that the surface morphology and microstructure of materials also can influence biological properties [6,26,30], because the surface roughness and peak-to-valley values of Ti-HA composites, which had better biological activity, were always higher than those of pure Titanium samples [10].

**Figure 13:**
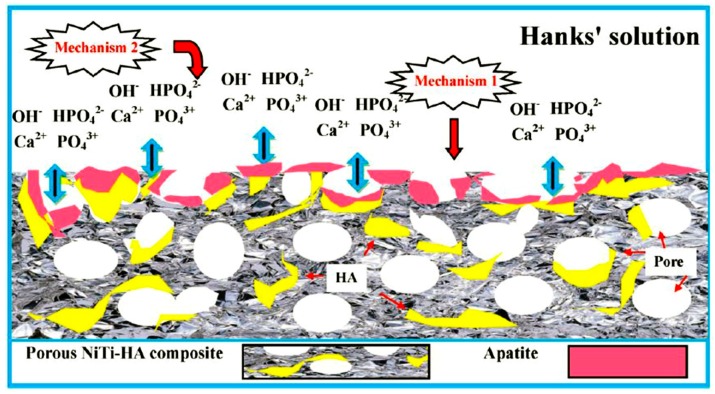
Schematic illustration of the formation mechanism of bone-like apatite layer on the surface of porous NiTi-HA composites [6].

It was found that the complete flake crystal Calcium-Phosphorus layers on the surface of NiTi-3% HA composites prepared by spark plasma sintering in Hanks’ solution have two main formation mechanisms (Figure 13) [6]: (1) The porous structure increased contact areas between the composite and simulated body fluids, resulting in the deposition of a large number of Calcium ions and Phosphate ions around pore walls. The larger contact areas provided sites for the nucleation and growth of apatite layer; (2) A large number of Ca^+2^, PO_4_^−3^, and HPO_4_^−2^ generated by reactions of HA entered the simulated body fluid and reacted with free ions, which further promoted the formation of calcium phosphate layers. The researchers found that nanoscale materials have a higher osteoblast adhesion rate and can promote osteoblast function, which is beneficial to tissue growth after transplantation [1,83,92]. Balbinotti et al. revealed that the nano-HA has smaller agglomeration and more uniform distribution in the composite, which results in the formation of more uniform Ca-P deposition in the simulated body fluid [89]. Although there were complex reactions between HA and Titanium during high temperature sintering, the formation of Ti_2_O, CaO, etc., also promoted the nucleation of apatite and increased biological activity of composites [21]. In addition, the bioactivity of composites can also be improved by some subsequent treatments, such as hydrothermal treatment [93].

In addition, the in vivo bioactivity and micro-mechanical properties at the interface between biomaterials and bone tissue were also analyzed [74,94,95,96]. Some researchers found that more new bone appears between the surface of Ti-HA composite with higher content of Titanium and the bone tissue during the initial stage after transplantation. After being transplanted for 6 months, all of the Ti-HA composites formed a chemical bone-bonding interface with the host bone through apatite layer [94]. However, some researches mentioned that the presence of titanium will reduce the osseointegration of HA-20% Ti composite implanted in the skull of New Zealand white rabbits at the initial stage of transplantation [74]. In fact, it was revealed that the reaction products of HA in HA-Ti composites dissolve faster than HA and can produce more Ca^2+^ and PO_4_^3−^, which will lead to the faster healing at the defectsite after transplantation. Moreover, the presence of HA and its products contributed to the formation of apatite layer between implant and bone tissue, and the presence of TiO_2_ on the surface of Titanium also provided more nucleation sites for the formation of apatite, which is beneficial to the increase of bonding strength between implant and bone tissues [74]. In addition, Alzubaydi et al. studied the biomechanical properties of HA, Partially Stabilized Zirconia (PSZ), and 50% HA + 50% PSZ coating on Titanium alloy surface [95]. Similarly, results also showed that as the time of implantation increases, the force needed to unscrew implant from the bone also gradually increases, which results in the improving of interfacial shear strength. Therefore, this, in turn, will promote stress transfer from implant to the bone and cause uniform distribution of stress between the bone and the implant. After 2 weeks of transplantation, the highest torque value (13.122 N·cm) was needed to unscrew 50% HA/50% PSZ-Ti composite coating implant from the bone tissue compared with that of Titanium alloy, HA coating, and PSZ coating (mean torque values 12.375, 10.875 and 8.87 N·cm, respectively). Moreover, the bonding strength between the implant and the bone tissue continued to increase due to the increase of bone implant contact degree and bone maturity degree during healing and remodeling [95]. Shiraishi et al. mentioned that the push-in test values of Ti and Ti-29Nb-13Ta-4.6Zr (TNTZ) alloy coated by the amorphous calcium phosphate (ACP) were significantly higher than those of Ti and TNTZ alloy after 8 weeks. This indicated that the osseointegrations of TNTZ alloy and Ti are improved by being coated with ACP, because bone tissue has a better resorption rate for ACP, which promotes the interface bonding between implant and bone tissue [96].

## 5. Conclusions

As a type of biomedical transplant materials, Titanium alloy/HA coated materials have problems with interfacial bonding between substrate and coating, while uncoated composites have some shortcomings such as complex reactions and shielding phenomena. Although the addition of nano-carbon and some metal oxides can improve the properties of Titanium alloy/HA composites to some extent, it is still challenging to achieve uniform distribution and integrity of the additive phases. The differences in the preparation methods and treatment techniques of Titanium alloy/HA composites affect the interface morphology and interfacial reactions due to their respective characteristics (temperature, thermal diffusion, etc.). In coating materials or uncoated composites, chemical reactions occur at their interfaces due to the high temperature decomposition of HA and its chemical reactions with Ti or other phases during the preparation. Mechanical bonding, caused by various factors such as cold welding and thinning, was one type of interfacial bonding in materials. Furthermore, the interfacial reactions between Ti and HA can be inhibited by adjusting the preparation process parameters. Due to its low thermal expansion coefficient and unique interface reactions, the bonding strength between coating and substrate is increased, and the residual interfacial stress is decreased when TiO_2_ or ZrO_2_ layers are added to the coating. The mechanical properties of Titanium alloy/HA composites are improved by adding nano-carbon, Fe, Sn, and other strengthening phases due to the mechanism of grain refinement strengthening, enhanced phase strengthening, solid solution strengthening, crack deflection, and drawing of graphene. In the future, it is necessary to construct the synergistic mechanisms of strengthening and biological activity, which will provide a guideline for the design of coating materials, monolithic materials, and gradient materials to control and regulate the material microstructure.

## Figures and Tables

**Table 1:** Comparison of the mechanical properties of Titanium and Titanium/HA biomaterials.

Process	Materials	Young’s Modulus	Vicker Hardness	Source
-	cp-Ti	100 GPa	-	[1]
Hot-pressing	Ti-20% HA	102.6 GPa	3.41 GPa	[1]
Hot-pressing	HA-20% Ti	75.91 GPa	3.13 GPa	[1]
Hot-pressing	cp-Ti	-	217 ± 1.19	[10]
Hot-pressing	Ti-30% nHA	-	383.8 ± 1.13	[10]
Plasma-sprayed coatings	HA/Ti	57.4 ± 5.3 GPa	3.9 ± 0.4 GPa	[11]
